# Adipose-Derived Stem Cell Exosomes Promote Scar-Free Healing of Diabetic Wounds via miR-204-5p/TGF-*β*1/Smad Pathway

**DOI:** 10.1155/sci/6344844

**Published:** 2025-02-19

**Authors:** Peijun Song, Qiu Liang, Xiuyu Ge, Danlian Zhou, Mei Yuan, Weiwei Chu, Jing Xu

**Affiliations:** ^1^Department of Plastic Surgery and Burn, The First Affiliated Hospital of Bengbu Medical University, Bengbu City 233000, Anhui Province, China; ^2^Department of Plastic Surgery, The Affiliated Hospital of Yangzhou University, Yangzhou City 225000, Jiangsu Province, China; ^3^Anhui Key Laboratory of Tissue Transplantation, Bengbu Medical University, Bengbu City 233000, Anhui Province, China

**Keywords:** adipose-derived stem cell, exosomes, fibroblasts, miR-204-5p, wound healing

## Abstract

Numerous researches have demonstrated the therapeutic potential of adipose-derived stem cell exosomes (ADSC-Exos) in promoting wound healing. In this study, we aimed to investigate the impact of ADSC-Exos on diabetic wound fibroblasts and elucidate its possible mechanisms. CCK-8, Edu, cell scratch, and Transwell tests were used to evaluate the function of ADSC-Exos on rat skin fibroblasts (RSFs) in high-glucose (HG) medium. The targeting effect of ADSC-Exo-derived microRNA (miRNA) and TGF-*β*1 was assessed using bioinformatic analysis and then confirmed with western blot and dual luciferase reporter assays. ADSC-Exos, miR-204-5p mimic, and anti-miR-204-5p mimic were used to stimulate RSFs, and the levels of TGF-*β*1/Smad pathway were analyzed by western blot. In vivo, digital photo and tissue section staining were used to evaluate the therapeutic effect of ADSC-Exos on diabetic wounds. The data showed that ADSC-Exos enhance the proliferation and migration of fibroblasts under HG conditions, reduce excessive myofibroblast differentiation and collagen deposition, and promote scarless healing of diabetic wounds. Additionally, miR-204-5p in ADSC-Exos targets TGF-*β*1 to inhibit p-Smad2/3, Col I, and alpha-smooth muscle actin (*α*-SMA), thereby reducing fibrosis. These findings suggest that ADSC-Exos have potential prospects for promoting diabetic wound healing.

## 1. Introduction

The skin, as the body's largest organ, contains various types of cells and serves as the primary protective barrier for the body. The process of healing skin wounds involves four interconnected stages: hemostasis, inflammation, proliferation, and remodeling [1]. Dysfunction and stagnation in the process can result in suboptimal wound healing outcomes, such as nonhealing wounds and pathological scarring. Diabetic ulcer is a prevalent and severe complication of diabetes, known for their challenging healing process and high incidence of disability. The common causes of delayed wound healing and scarring in diabetes mellitus are persistent inflammation, epithelial dysfunction, microvascular dysfunction, and peripheral neuropathy induced by hyperglycemia [2–4]. Researches have shown that approximately 30% of diabetes patients face the issue of diabetic foot ulcers, with an amputation rate as high as 20% [4, 5]. Although there are various treatment methods in clinical practice, such as medication, surgery, and wound dressings, these existing methods have been proven to be insufficient in effectively addressing the slow healing and high disability rate of diabetic wounds [6–8]. Therefore, it is valuable to explore new methods for diabetic wound healing. Not only can it alleviate the suffering of diabetic patients and improve their living quality, but it also reduces medical expenditure.

It has been proven that mesenchymal stem cells (MSCs) play a significant role in promoting wound healing. Compared to other MSCs, adipose-derived stem cells (ADSCs) have the advantages of abundant source, easy accessibility, and minimally invasive procedures. As a result, they hold tremendous potential for development in regenerative medicine and tissue engineering [9]. Researches have shown that the exosomes released by ADSCs play a crucial role in the therapeutic effects through their paracrine function [10]. Exosomes are a type of double-layered membrane extracellular vesicles with a diameter ranging from 40 to 160 nm. These vesicles contain various proteins, lipids, and ribonucleic acids. In comparison to ADSCs, ADSC exosomes (ADSC-Exos) exhibit similar biological activities but possess smaller sizes and better biocompatibility. As a result, they can more effectively traverse cell membranes to facilitate intercellular signaling, thereby regulating a range of cellular functions [11]. Previous researches showed ADSC-Exos released various bioactive, such as proteins and ribonucleic acids, which participated in multiple stages of wound healing. This process enhances wound healing by regulating immunity, neovascularization, epithelial cells, and tissue remodeling. Ultimately, it accelerates wound closure and reduces scar formation [12–14]. In conclusion, ADSC-Exos have enormous potential for the treatment of chronic wounds.

Fibroblast is a type of key cell in the wound healing process, especially in promoting re-epithelialization and tissue remodeling [15]. Previous studies have demonstrated that high-glucose (HG) concentration leads to chronic inflammation, oxidative stress, and cellular senescence and consequently impairs the function of fibroblasts, resulting in delayed wound healing [16–18]. However, researchers have found that ADSC-Exos can be taken in by dermal fibroblasts. Consequently, ADSC-Exos can boost the proliferation and migration of fibroblasts and regulate collagen remodeling. Ultimately, this contributes to the promotion of wound healing [19]. Fibroblasts undergo differentiation into myofibroblasts, which subsequently facilitate wound contraction and remodeling of the extracellular matrix (ECM). Alpha-smooth muscle actin (*α*-SMA) is currently the most widely used molecular marker for myofibroblasts [20]. Finally, unnecessary fibroblast cells gradually undergo apoptosis to prevent excessive fibrosis. Differing from the normal wound healing process, HG and oxidative stress can lead to sustained activation or dysregulation of apoptosis in myofibroblasts, resulting in excessive ECM formation and pathological scarring. Therefore, maintaining the balance of myofibroblast differentiation is crucial for proper wound healing [21–23]. The TGF-*β*1/Smad pathway is a classic activation pathway for myofibroblast differentiation and plays a major role in fibrosis [22]. There is evidence from research that ADSC-Exos inhibited *α*-SMA expression by downregulating the ratio of TGF-*β*1/TGF-*β*3, thus preventing fibroblasts from differentiating into myofibroblasts and reducing scar thickness and length [24]. In conclusion, ADSC-Exos can improve the proliferation and migration of fibroblasts, and also prevent fibroblasts from differentiating into myofibroblasts. The mechanism by which ADSC-Exos affect diabetic wounds deserves further exploration in academic research.

MicroRNA (miRNA) is an endogenous noncoding ribonucleic acid that can degrade target mRNA or inhibit its translation. MiRNA mimics and inhibitors are expected to become new therapeutic targets [25]. Research has shown that diabetes or its complications can lead to changes in miRNA, therefore making miRNA a promising therapeutic target [26]. This highlights the importance of utilizing miRNA as a potential treatment for diabetes and its associated conditions. The upregulation of miRNA in ADSC-Exos has also attracted widespread attention from scientists. Through comprehensive genetic analysis, they have revealed the significant roles of these miRNAs in processes such as cellular senescence, tumorigenesis and progression, and inflammation [27–29]. Choi et al. [30] performed a gene microarray analysis of ADSC-Exos and revealed that they are enriched with miRNAs that promote regenerative functions. These miRNAs work by suppressing genes such as NPM1, PDCD4, CCL5, and NUP62, thereby contributing to the proliferation and fibrosis of dermal fibroblasts [30]. In general, miRNA may play a role in wound healing and scar formation by alleviating cellular senescence, inflammatory response, epithelial cell function, etc. In this study, we established a cell model by treating rat skin fibroblasts (RSFs) with HG (33 mmol/L) to simulate the diabetic extracellular environment. At the same time, we excised full-thickness skin from type 2 diabetic rats' backs to establish an animal model. The ADSC-Exos were used to treat both cells and animal models, with an equal volume of PBS used as the control group. Through bioinformatics analysis, we have also discovered that miRNA in ADSC-Exo can inhibit the TGF-*β*1/Smad signaling pathway by targeting TGF-*β*1. Therefore, we hypothesized that ADSC-Exos can improve wound healing and reduce scar formation by delivering miRNA.

## 2. Materials and Methods

### 2.1. Ethical Statement

The SD rats were purchased from the Shandong Experimental Animal Center. The animal experimental procedures in this study followed the “Guidelines for the Care and Use of Laboratory Animals.” Animal experiments were approved by the Ethics Committee of Bengbu Medical University (No: 289. 2022).

### 2.2. Cultivation of Fibroblasts From Rat Skin

The SD rats were anesthetized with 30 mg/kg of pentobarbital sodium before the operation began. Skin tissue is taken from fetal rats, thoroughly washed with physiological saline, and excess fat tissue under the dermis is removed. RSFs were isolated using a combination of 0.1% neutral protease and 0.2% I-type collagenase. The complete growth medium consists of 89% DMEM (Gibco, USA), 10% FBS (Gibco, USA), and 1% penicillin/streptomycin (Biosharp, China). The culture environment is set at 37°C with 5% CO_2_ in a CO_2_ incubator.

### 2.3. Cultivation and Identification of ADSCs

The adipose tissue, which was obtained from the inguinal region of SD rats and cut into 1 mm, was digested in a tube at 37°C for 60 min using 0.2% I-type collagenase (Solarbio, China). Stop digestion with an equal volume of 10%FBS DMEM. After centrifugation at 1000x*g* for 10 min, mix the precipitate with complete culture medium, then filter through a 200-mesh sieve. Subsequently, centrifugation at 1000x *g* for 5 min, the cell pellet was resuspended in a complete medium for cultivation at 37°C with 5% CO_2_. Cell culture was conducted using adipogenic, osteogenic, and chondrogenic induction media according to the instructions (Procell, China). After 21 days, cells were stained with Oil Red O, Alizarin Red, and Safranin O. Collect the third generation of ADSC for flow cytometry, digest the cells with trypsin, block with 1% BSA for 30 min, incubate with fluorescent antibodies CD29 (102215), CD90 (206105), and CD45 (202207) from Biolegend (USA), CD34 (sc-7324) from Santa Cruz (USA), analyzing by a flow cytometer (Beckman, CytoFLEX, USA).

### 2.4. Isolation and Identification of ADSC-Exos

The steps for separating exosomes were shown in the previous experiments [31]. Cultivate ADSCs with a fusion degree of 70%–80% in serum-free MSC media (UR51120, Umibio, China). After 48 h, collect the cell supernatant and perform centrifugation in steps at 4°C: first at 300x*g* for 10 min, and 2000x *g* for 20 min, 10,000x *g* for 30 min. Then, filter through a 0.22 μm filter. Subsequently, ultracentrifuge at 100,000x *g* for 70 min to obtain the precipitate as exosomes. Finally, resuspend the exosomes in PBS and store them at −80°C.

The concentration and size distribution of ADSC-Exos were analyzed by transmission electron microscopy (TEM) and nanoparticle tracking analysis (NTA). Surface markers of ADSC-Exos, such as CD9 (AF5139), CD63 (AF5117), TSG101 (DF8427), and Calnexin (AF5362) from Affinity (China) were detected through western blotting (Specific steps are described in 14). Using a BCA protein assay kit (Beyotime, China) to determine the total protein concentration of ADSC-Exos.

### 2.5. Cell Uptake Exosomes Experiment

According to the instructions, ADSC-Exos were labeled with fluorescent dye PKH26 (MKBio, China). RSFs were seeded in cell climbing slices and cocultured with ADSC-Exos in a serum-free medium for 24 h. The cells were fixed with 4% paraformaldehyde, stained for cytoskeleton with phalloidin, and for nuclei with DAPI. Images were captured using a fluorescence microscope (ZEISS, Observer Z1, Germany) for observation.

### 2.6. CCK-8 Experiment

The proliferation of RSFs was assessed using CCK-8 (Biosharp, China). About 100 μL of 5 × 10^3^ RSFs were seeded into a 96-well plate. HG was set at a concentration of 33 mmol/L, while normal control at 5 mmol/L. The experimental groups were as follows: control, HG + PBS, HG + ADSC-Exos. The cells were treated with 100 μg/mL ADSC-Exos for 24, 48, and 72 h, while control and HG + PBS were treated with an equal volume of PBS. Subsequently, 10 μL of CCK-8 reagents was added to each well and incubated at 37°C for 2 h. The absorbance at the wavelength of 450 nm was measured using a microplate reader (Thermo, USA). The cell proliferation rate is proportional to the 450 nm OD value.

### 2.7. Edu Staining

Transfer RSFs from the logarithmic growth phase to the 12-well plate. The fusion rate of cells reaches 70%–80% and staining is carried out with an Edu assay kit (Beyotime, China). Incubate cells with Edu working solution at 37°C for 2 h. Then, treat with 4% paraformaldehyde for 15 min. Next, permeabilized with 0.3% Triton x –100 for 10 min, followed by a 30-minute incubation in Edu reaction solution in the dark. Finally, stain with Hoechst 33,342 for 10 min. The principle of EdU staining is that EdU (5-ethynyl-2′-deoxyuridine nucleoside) binds to DNA during cell proliferation, forming an EdU-DNA complex. By detecting the signal intensity of this complex, cell proliferation can be determined. Cell proliferation rate = Edu cell number/total cell number x100%.

### 2.8. Cell Migration Experiment

Cell Scratch Assay: Treat RSFs with serum-free DMEM for 12 h. Cell proliferation was inhibited by 10 μg/mL colchicine. A line was scratched on the bottom of the plate using a 200 μL pipette tip. About 100 μg/mL ADSC-Exos were added, and PBS for the control group. After 24 h, images were captured and observed under a microscope. Percentage of wound healing = initial scratch area− final scratch area/initial scratch area x100%.

Transwell Assay: Transwell chambers (Corning, USA) with a pore size of 8 μm were placed in a 24-well plate. A total of 200 μL of cell suspension diluted in serum-free culture medium was added to the upper chamber, while 500 μL of complete culture medium, supplemented with ADSC-Exos (100 μg/mL) or PBS, was added to the lower chamber. After 24 h, staining with 0.2% crystal violet solution. Microscope pictures were taken, and the number of purple cells in the pictures were counted and statistically analyzed.

### 2.9. Analysis of Bioinformatics

The sequencing data GSE92313 from the Gene Expression Omnibus (GEO) database was analyzed to identify miRNA from rat-derived ADSC-Exos. The miRNA target genes were analyzed and predicted through the miRWalk website (www.uni-heidelberg.de).

### 2.10. RNA Extraction and RT-qPCR

Total RNA was extracted using TRIzol Reagent (Sangon Biotech, China). For the analysis of miR-204-5p expression in ADSC-Exos, qPCR was performed using a One-Step RT-qPCR Kit (Sangon Biotech, China) on a QuantStudioTM3 system (Thermo Fisher Scientific, USA). The relative expression level of the target gene was calculated using the 2^−∆∆Ct^ method.

Rno-mir-204 (95435-1)-F, 5′-TAAGGTGTCGGAGAATCAAGATG-3′,

Rno-mir-204 (95435-1)-R, 5′-TGTCACCCTTGTCTTGGGAAAG-3′.

U6-F, 5′-CTCGCTTCGGCAGCACA-3′,

U6-R, 5′-AACCGCTTCACGAATTTGCGT-3′.

### 2.11. Plasmid Transfection Experiment

When the cell fusion rate reaches 80%, according to the manufacturer's instructions, miR-204-5p mimic, anti-miR-204-5p, and their control plasmids were transfected into RSFs, and the plasmid was purchased from Genechem Co (China). LipofectamineTM 2000 from Invitrogen (USA) was used to promote plasmid transfection. MiR-204-5p mimic sequence: 5'-CCTTTGTCA-3'. Anti-miR-204-5p sequence: 5'-TGACAAAGG-3'. MiR-NC was the GV716 vector, and anti-miR NC was the GV249 vector; the two vectors were purchased from Genechem Inc., Shanghai, China.

### 2.12. Dual-Luciferase Reporter Assay

PCR amplification of miR-204-5p was performed. TGF-*β*1-wt and TGF-*β*1-mut constructs were generated using the pGL3 basic vector. Transfection of TGF-*β*1-wt, TGF-*β*1-mut, and miR-204-5p mimic or control group was carried out with NIH 3T3 cells. After 48 h, the luciferase activity was measured using the Dual-Luciferase Reporter Assay System (Promega, USA).

### 2.13. Animal Model of Diabetic Wound

The 8-week-old male SD rats, weight (280 ± 20 g), were randomly divided into the healthy group, diabetes control group, and diabetes ADSC-Exos group (*n* = 6). The rats were fed a high-fat diet for 4 weeks, followed by intraperitoneal injection of 35 mg/kg Streptozocin (STZ, 60256ES80, YEASEN, China). Fasting blood glucose greater than 11.1 mmol/L after 7 days was considered a diabetic rat. The surgery started 2 weeks after the STZ injections. Two full-thickness skin wounds with a diameter of 10 mm were created on both sides of the rat's back. ADSC-Exos (100 μg/mL) and PBS 1 mL were injected subcutaneously into the wounds on days 0, 3, 7, and 10 for each group. Wound photographs were taken on days 0, 3, 7, 10 and 14. Finally, a circular skin sample with a diameter of 10 mm was removed from the wound for staining and western blot.

### 2.14. Western Blot Experiment

Cell or skin tissue was lysed using RIPA lysis buffer (Biosharp, China), followed by 30 min of ice incubation and centrifugation at 12,000 rpm for 15 min at 4°C. The precipitate was then quantified for total protein content using the BCA protein assay kit (Beyotime, China). Denaturation of the proteins was achieved by boiling with sample loading buffer for 5 min, followed by SDS–PAGE electrophoresis (80 V for 30 min; 120 V for 60 min) and membrane transfer (200 mA for 90 min) onto a PVDF membrane. The membrane was blocked with 5% nonfat milk at room temperature for 1 h, then probed with primary antibodies (1:1000) overnight at 4°C and secondary antibodies (1:5000) at room temperature for an additional hour. Visualization was performed using Bio-RAD ChemiDoc. TGF-*β*1(BF8012), *α*-SMA (AF1032), Collagen I (AF7001) from Affinity, China. Smad2/3(8685 s), p-smad2/3(8828 s) from CST, USA. GAPDH (GB11002-100) and *β*-actin (GB11001-100) from Servicebio, China. Image J software was used to determine the gray values of the protein bands. Protein semi-quantification = average gray value of target protein bands/average gray value of control protein bands x100%.

### 2.15. H&E Staining

Paraffin sections were deparaffinized in xylene and hydrated in gradient alcohol, followed by hematoxylin staining of nuclei for 10 min, washing in tap water for 1 min, differentiation with 1% hydrochloric acid for 5 s, washing in water and transblue treatment with 1% ammonia for 3–4 s and washing in water for 1 min. Finally, the samples were processed in eosin solution for 2–5 min. After completion of the above steps, the samples were dehydrated again, visualized, and encapsulated. After the above steps, the samples were dehydrated, visualized, and encapsulated again. The width of the scar in HE sections and the thickness of the epithelial cells were calculated using Image J software and analyzed using GraphPad Prism.

### 2.16. Masson Staining

After dewaxing and hydration, the nuclei were stained with hematoxylin for 5 min, differentiated with 1% hydrochloric acid for 5 s, counterstained with 1% ammonia, stained with Reichhorn red for 5 min, and finally stained with phosphomolybdic acid for 5 min and phenylamine blue for 5 min. Collagen is shown in blue. The density of the blue areas was calculated using Image J software, and the results were analyzed.

### 2.17. Immunofluorescence Staining

The waxes were removed and hydrated by 3% hydrogen peroxide at room temperature and, incubated for 10 min, then washed again with PBS buffer. The samples were placed in citrate buffer and heated in a microwave oven at high power for 30 min and allowed to cool to room temperature. The goat serum was incubated for 30 min at room temperature, then incubated with primary antibody overnight at 4°C, rinsed with PBS, and incubated with fluorescent secondary antibody (1 h at room temperature). Primary antibody TGF-*β*1(BF8012), *α*-SMA (AF1032), Collagen I (AF7001) from Affinity, China. Fluorescent secondary antibody Phalloidin-iFluor 488 (ab176753) Phalloidin-iFluor 647 (ab176759) Phalloidin-iFluor 555 (ab176756) from Abcam, USA. The samples were then reacted with DAPI for 15 min, blocked, and observed under a microscope. The mean intensity of fluorescence was analyzed using Image J.

### 2.18. Statistical Analysis

All data were analyzed using GraphPad Prism 9.0. The data are presented as mean ± standard deviation. The comparison between two groups was conducted using *t*-test, while multiple group comparisons were performed using analysis of variance (ANOVA) followed by Tukey's post hoc test. *⁣*^*∗*^*p*  < 0.05, *⁣*^*∗∗*^*p*  < 0.01, *⁣*^*∗∗∗*^*p*  < 0.001.

## 3. Results

### 3.1. Identification of ADSCs and ADSC-Exos

**Figure 1:**
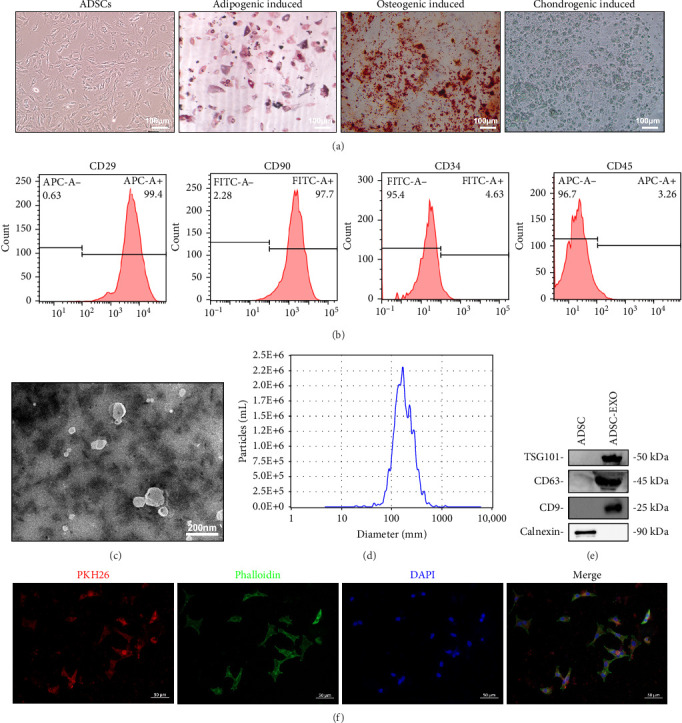
Identification of ADSCs and ADSC-Exos. (A) ADSCs and trilineage differentiation results (scale bar = 100 μm). (B) Flow cytometry analysis of markers on ADSCs, including CD29, CD90, CD34, and CD45. (C) TEM image showing the morphology of ADSC-Exos (scale bar = 200 nm). (D) NTA result of ADSC-Exos. (E) Western blot evaluation of TSG101, CD63, CD9, and Calnexin proteins. (F) Fluorescence inverted microscope observation of PKH26-labeled ADSC-Exos (scale bar = 50 μm). ADSC-Exos, adipose-derived stem cell exosomes; ADSCs, adipose-derived stem cells; NTA, nanoparticle tracking analysis; TEM, transmission electron microscopy.

After isolation and cultivation of ADSCs from the adipose tissue of rats for 72 h, observation was conducted using an inverted microscope. The cells adhered to the bottom of the culture dish, presenting a spindle-shaped and polygonal morphology, with nuclei appearing relatively large and clearly visible (Figure 1A). The initially isolated ADSCs were contaminated by blood cells, and as the cells underwent passages, the blood cell population gradually decreased. We chose to characterize and identify the 3rd generation of ADSCs for further analysis. After 7 days of adipogenic induction culture, the ADSCs began to exhibit well-defined transparent lipid droplets. Subsequently, these droplets gradually increased in size and aggregated, causing a transition in cell morphology from spindle-shaped to round. Upon Oil Red O staining on day 21, the cells displayed a red coloration. After 10 days of osteogenic induction, white sandy granular precipitates can be observed with cell fusion into sheets, and the boundaries are not clear. After staining with alizarin red on day 21, the formation of brown calcium nodules can be observed. After 21 days of chondrogenic induction, white cartilage microspheres were observed, and alcian blue staining revealed the presence of blue cartilage particles (Figure 1A). Flow cytometry analysis revealed that the ADSCs expressed high levels of MSCs markers CD29 (99.4%) and CD90 (97.7%), while hematopoietic stem cell markers CD34 (4.63%) and CD45 (3.26%) were expressed at low levels (Figure 1B). These results indicate that the ADSCs possess the characteristics of MSCs and multilineage differentiation ability.

Observed under TEM, ADSC-Exos exhibited a cup-shaped or circular morphology with clear edges (Figure 1C). The size of ADSC-Exos was measured using a Nanometer particle tracking instrument, and the results showed that the average diameter of the particles was 165.8 nm, with a concentration of ~6.1E + 10 particles/mL (Figure 1D). The western blot experiment detected positive expression of TSG101 (tumor susceptibility gene 101) and CD63, CD9 (transmembrane proteins), and negative expression of Calnexin (endoplasmic reticulum protein) (Figure 1E). The above results are consistent with the characteristics of exosomes.

To investigate whether ADSC-Exos can be internalized by dermal fibroblasts, we labeled ADSC-Exos with PKH26 and added them to the culture medium of RSFs at a final concentration of 100 μg/mL. After coculturing for 24 h, ADSC-Exos were found inside RSFs and distributed around the nucleus (Figure 1F). The results indicate that ADSC-Exos can be uptake by RSFs.

### 3.2. ADSC-Exos Repaired Fibroblast Damage Induced by HG In Vitro

**Figure 2:**
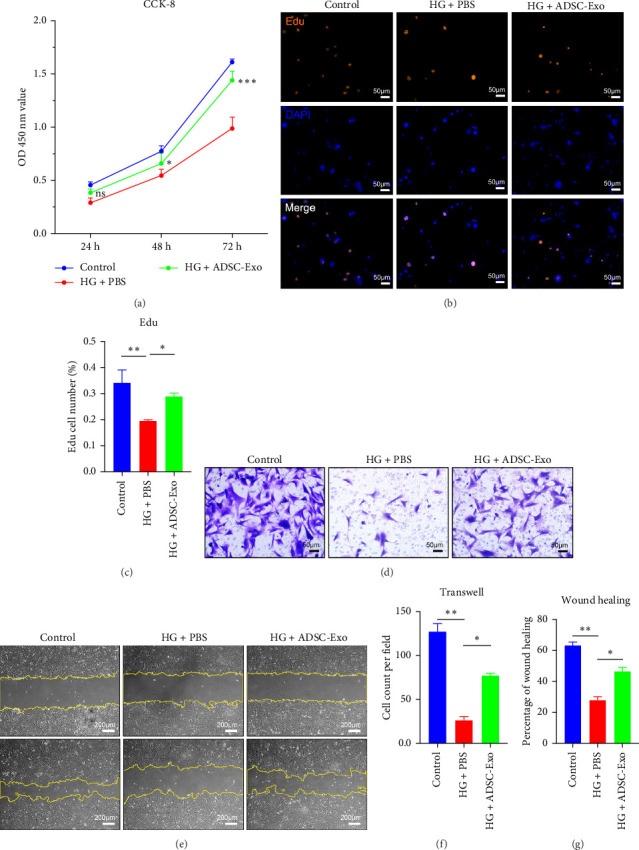
ADSC-Exos promoted proliferation and migration of RSFs. (A) Evaluate RSFs proliferation using CCK-8 assay. (B) Detect RSFs proliferation with Edu experiment. (C) Statistical analysis of Edu-positive cells. (D) Measure RSFs migration using Transwell chamber assay. (E) Assess RSFs migration through scratch experiment. (F) Statistical analysis of Transwell assay. (G) Statistical analysis of wound healing ratio in the scratch experiment. ns: *p* > 0.05, *⁣*^*∗*^*p* < 0.05, *⁣*^*∗∗*^*p* < 0.01, and *⁣*^*∗∗∗*^*p* < 0.001. ADSC-Exos, adipose-derived stem cell exosomes; RSFs, rat skin fibroblasts.

According to reports, high concentrations of glucose may cause damage to the function of fibroblasts. We established an HG (33 mmol/L) culture medium. Experimental groups were set as follows: control group, HG control group (HG + PBS), and HG ADSC-Exos treatment group (HG + ADSC-Exo). A final concentration of 100 μg/mL ADSC-Exos was added to the HG + ADSC-Exo group, while an equal amount of PBS was added to the control group and HG + PBS group. In both CCK8 and Edu experiments, it was observed that the proliferation activity of the HG + PBS group was significantly lower than that of the control group. In the CCK8 experiment, there was no significant difference in the OD value at 24 h between the HG + ADSC-Exo group and the HG + PBS group. However, from 48 to 72 h, the OD value of the HG + ADSC-Exo group showed a significant increase (*p*  < 0.05) (Figure 2A). In the Edu experiment, the number of Edu-positive cells in the HG + PBS group decreased significantly compared to the control group. After ADSC-Exos treatment, Edu-positive cells increased (*p*  < 0.05) (Figure 2B,C). The results of the Transwell migration experiment and cell scratch test showed that ADSC-Exo significantly enhanced the migration ability of HG-induced RSFs (*p*  < 0.05) (Figure 2D–G). The above results indicate that ADSC-Exo can rescue the impaired proliferation and migration ability of HG-induced RSFs in vitro.

### 3.3. ADSC-Exos Promoted Scar-Free Wound Healing

**Figure 3:**
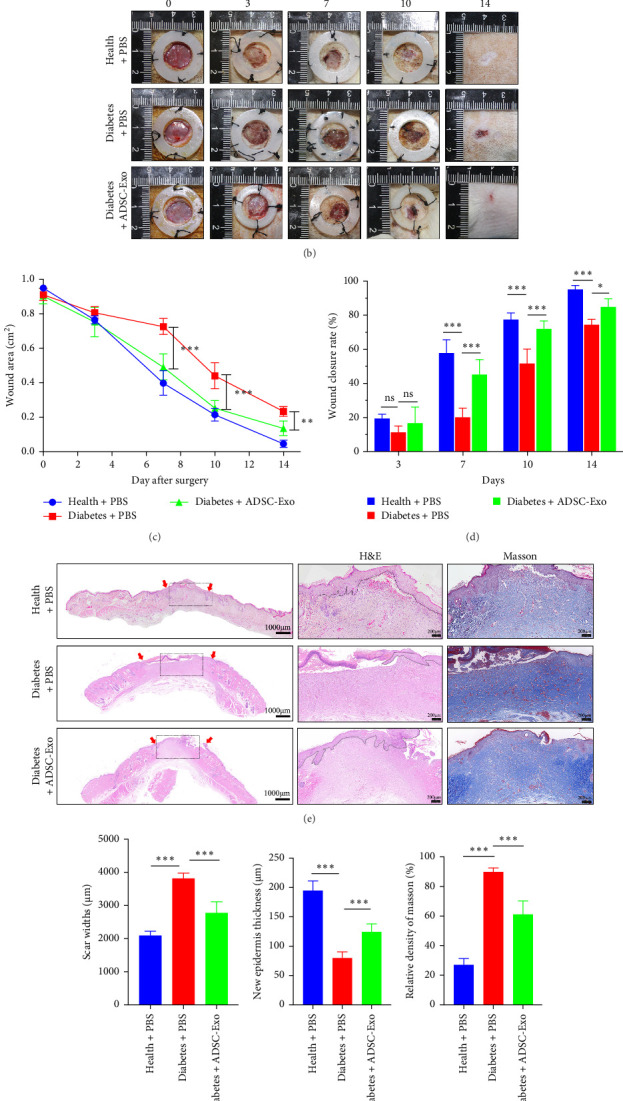
ADSC-Exos promote skin wound healing. (A) Animal experiment procedure. (B) Digital photo observation of rat skin wound healing. (C) Wound area statistics. (D) Wound closure rate statistics. (E) H&E and Masson staining. (F) Scar length statistics in H&E staining. (G) Epithelial thickness statistics in H&E staining. (H) Collagen fiber density statistics in Masson staining. ns: *p* > 0.05, *⁣*^*∗*^*p* < 0.05, *⁣*^*∗∗*^*p* < 0.01, and *⁣*^*∗∗∗*^*p* < 0.001. ADSC-Exos, adipose-derived stem cell exosomes.

In vitro experiments have shown that ADSC-Exos can enhance the proliferation and migration ability of fibroblasts induced by HG. In order to investigate the effect of ADSC-Exos on skin wound healing, we used a skin full-thickness defect model in STZ-induced diabetic rats and injected ADSC-Exos subcutaneously into wounds, with PBS as a blank control. The experimental procedure is shown in Figure 3A.

The digital photo results showed that the healing speed of diabetic wounds is significantly slower than that of healthy group wounds. Otherwise, ADSC-Exos can alleviate this phenomenon (Figure 3B). At 7, 10, and 14 days after surgery, the area of unhealed wounds in the diabetes + PBS group (0.73 ± 0.05, 0.44 ± 0.08, 0.23 ± 0.03 cm^2^) was larger than health group (0.40 ± 0.07, 0.21 ± 0.04, 0.05 ± 0.02 cm^2^). After treatment with ADSC-Exos, the wound area decreased (0.49 ± 0.08 cm^2^, 0.25 ± 0.04, 0.14 ± 0.04 cm^2^) (Figure 3C). The closure rates of the wounds in the health group were 58.04% ± 7.51%, 77.55% ± 3.81%, and 95.30% ± 2.19% on days 7, 10, and 14. In the diabetes group, closure rates significantly decreased to 20.28% ± 5.18%, 51.79% ± 8.32%, and 74.48% ± 3.09% on the same respective days. After ADSC-Exos treatment, the closure rates improved to 45.50% ± 8.46%, 71.97% ± 4.70%, and 85.08% ± 4.75% (Figure 3D). These results demonstrated that ADSC-Exos promoted the healing of diabetic wounds (*p*  < 0.05).

Assess the pathological condition of the wound using H&E staining. Compared to the healthy rats, the diabetic rats exhibited a reduced capacity for epidermal regeneration in wounds, immature granulation tissue, and longer scars. (Figure 3E). The scar length of the Diabetes + PBS group (3810.30 ± 163.41 μm) was significantly longer compared to the health group (2084.19 ± 135.90 μm). The results showed that ADSC-Exos reduced scar length (2768.35 ± 342.78 μm) (Figure 3F) (*p*  < 0.001). In the health group, the wound was completely covered by regenerated epithelial cells, and the epithelium was thicker (194.61 ± 16.57 μm). In the diabetes + PBS group, the wound had less and thinner epithelial coverage (80.12 ± 10.23 μm). ADSC-Exos significantly increased both the surface area and thickness of epithelial coverage in diabetic wounds (124.69 ± 13.27 μm) (*p*  < 0.001) (Figure 3G). In addition, the wound bed of the diabetes + PBS group showed increased granulation tissue, with more inflammatory cells and neovascularization. This indicates that on day 14, the untreated diabetic wound bed remains in the inflammation and granulation tissue proliferation phase, with a temporal and spatial overlap of tissue remodeling. However, the health group and Diabetes + ADSC-Exo group showed a reduction in inflammatory cells and granulation tissue, indicating a transition to the tissue remodeling phase.

### 3.4. Analysis of Fibrosis in Wound Scars

**Figure 4:**
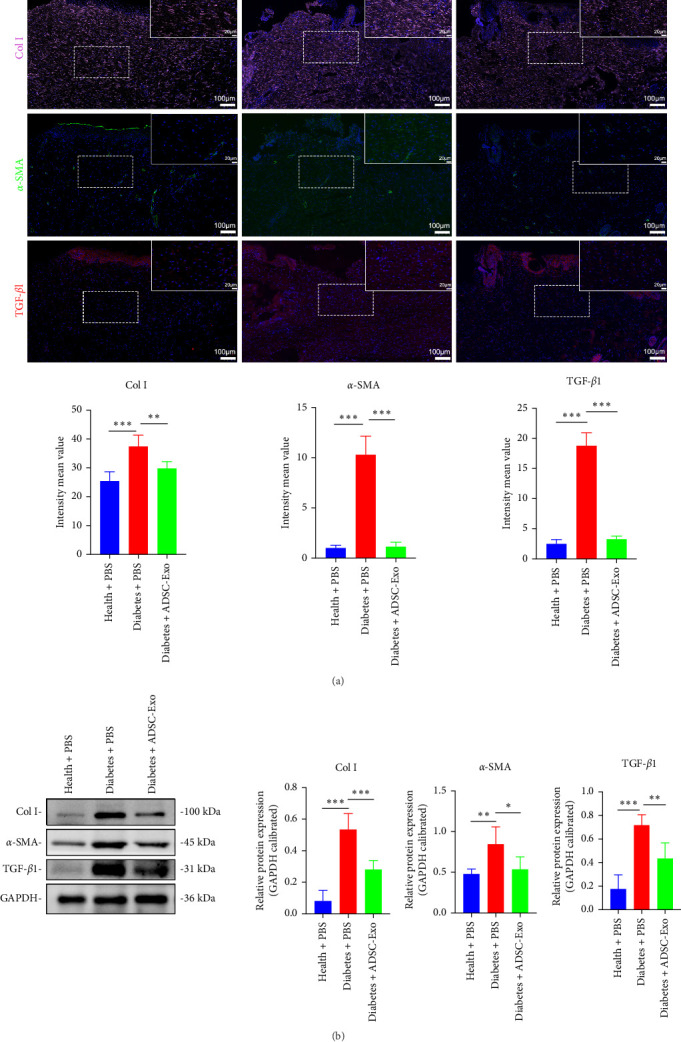
Analysis of fibrosis in wound scars. (A) Immunofluorescence staining of paraffin sections to assess the expression of Col I, *α*-SMA, and TGF-*β*1. (B) Western blot experiment to detect the expression of Col I, *α*-SMA, and TGF-*β*1. *⁣*^*∗*^*p* < 0.05, *⁣*^*∗∗*^*p* < 0.01, and *⁣*^*∗∗∗*^*p* < 0.001. *α*-SMA, alpha-smooth muscle actin.

To assess the fibrosis of the wound scar on day 14, we utilized Masson staining and immunofluorescence staining to examine the deposition of collagen fibers. In Masson staining, compared with the health group, the untreated diabetic wound had a higher density and more disordered arrangement of collagen fibers; ADSC-Exos reduces the density of collagen fibers (Figure 3E,H). Immunofluorescence staining was performed to detect the expression of collagen I, *α*-SMA, and TGF-*β*1 proteins. As shown in Figure 4, compared with the health group, collagen I was significantly deposited in diabetic wounds, while ADSC-Exos can reduce the deposition of collagen I (*p*  < 0.01). The generation of collagen I is closely associated with *α*-SMA, which is a marker for myofibroblasts. The observation of a large amount of *α*-SMA in diabetic wounds indicated a high degree of differentiation of fibroblasts into myofibroblasts. ADSC-Exos significantly reduced the differentiation of myofibroblasts (*p*  < 0.001). The expression of TGF-*β*1 is excessively activated in diabetic wounds, and ADSC-Exos can inhibit the activation of TGF-*β*1 (*p*  < 0.001) (Figure 4A). Meanwhile, the western blot experiment results demonstrated that the expression of collagen I, *α*-SMA, and TGF-*β*1 proteins in the diabetes control group was significantly higher than that in the health group. However, the treatment of ADSC-Exos in diabetic wounds was found to decrease the expression of these proteins (*p*  < 0.05) (Figure 4B). These results indicated that the degree of fibrosis is higher in the diabetes group, with more severe deposition of collagen I. ADSC-Exos can reduce the formation of collagen I and regulate tissue remodeling during the healing process, making it more similar to that of the health group.

### 3.5. ADSC-Exos Inhibited Scar Fibrosis by Targeting TGF-*β*1 via miRNA

**Figure 5:**
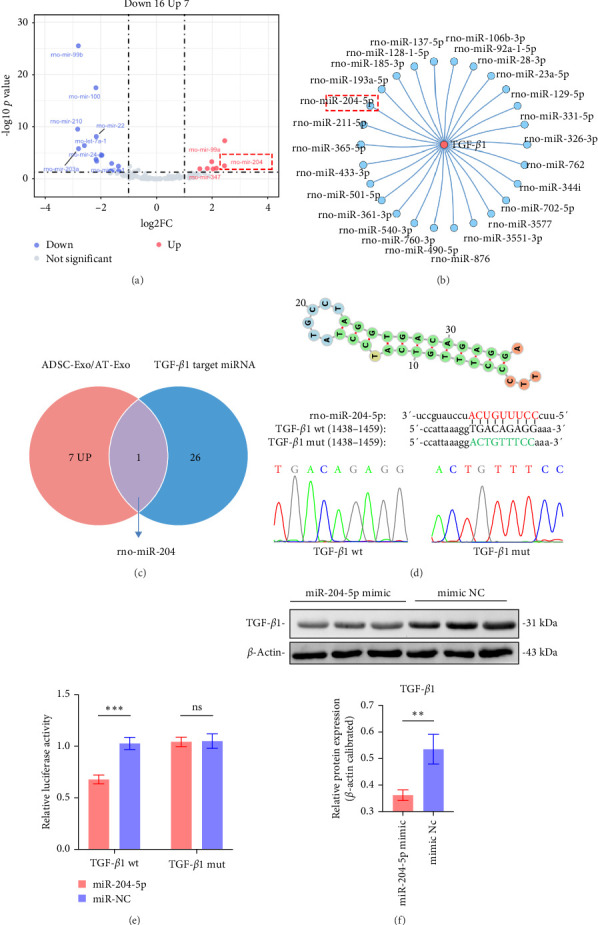
MiR-204-5p in ADSC-Exos targets TGF-*β*1. (A) Analysis of miRNAs in ADSC-Exos/AT-Exos from GSE92313. (B) Prediction of miRNAs targeting TGF-*β*1. (C) Rno-miR-204 is highly expressed in ADSC-Exos and targets TGF-*β*1. (D) Schematic diagram showing the binding sites of Rno-miR-204-5p and TGF-*β*1. (E) Dual-luciferase assay confirms the targeting of TGF-*β*1 by miR-204-5p. (F) Western blot validation demonstrates that transfection of RSFs with miR-204-5p inhibits TGF-*β*1 expression. ns: *p* > 0.05, *⁣*^*∗*^*p* < 0.05, *⁣*^*∗∗*^*p* < 0.01, and *⁣*^*∗∗∗*^*p* < 0.001. ADSC-Exos, adipose-derived stem cell exosomes; miRNAs, microRNAs; RSFs, rat skin fibroblasts.

miRNA in exosomes is one of the key active ingredients. In the previous results, we found that ADSC-Exos can inhibit the TGF-*β*1 signaling pathway and its downstream genes *α*-SMA and TGF-*β*1 expression. Therefore, we speculate that ADSC-Exos contain certain miRNAs that can target TGF-*β*1. We analyzed the miRNAs in rat ADSC-Exos reported by Zhang et al. [32] (GSE92313). Seven miRNAs were found to be highly expressed in ADSC-Exos (log2FC > 1, *p* < 0.05) (Figure 5A). By using miRWalk, we identified 26 miRNAs that target the 3′UTR region of TGF-*β*1, indicating their potential interaction with the TGF-*β*1 signaling pathway (Figure 5B). Based on the above results, it was found that rno-miR-204-5p is present in ADSC-Exos and simultaneously targets TGF-*β*1 (Figure 5C). Subsequently, the target genes of rno-miR-204-5p were predicted using the miRBase database, revealing its direct targeting of TGF-*β*1 mRNA (Figure 5D).

The results of the dual-luciferase report showed that TGF-*β*1 wt caused a decrease in luciferase activity, while TGF-*β*1-mut had no significant change. Therefore, TGF-*β*1 was confirmed as a direct target of rno-miR-204-5p (Figure 5E). Transfection of RSFs with miR-204-5p mimic and mimic NC, respectively, resulted in significant inhibition of TGF-*β*1 expression, as demonstrated by western blot analysis (Figure 5F). In vivo experiments also showed that local injection of ADSC-Exos in diabetic wounds reduced the levels of TGF-*β*1. These findings suggest that the process by which ADSC-Exos inhibit TGF-*β*1 may be achieved through direct targeting of TGF-*β*1 by miR-204-5p.

### 3.6. ADSC-Exos Regulated TGF-*β*1/Smad Signaling Pathway Through miR-204-5p

**Figure 6:**
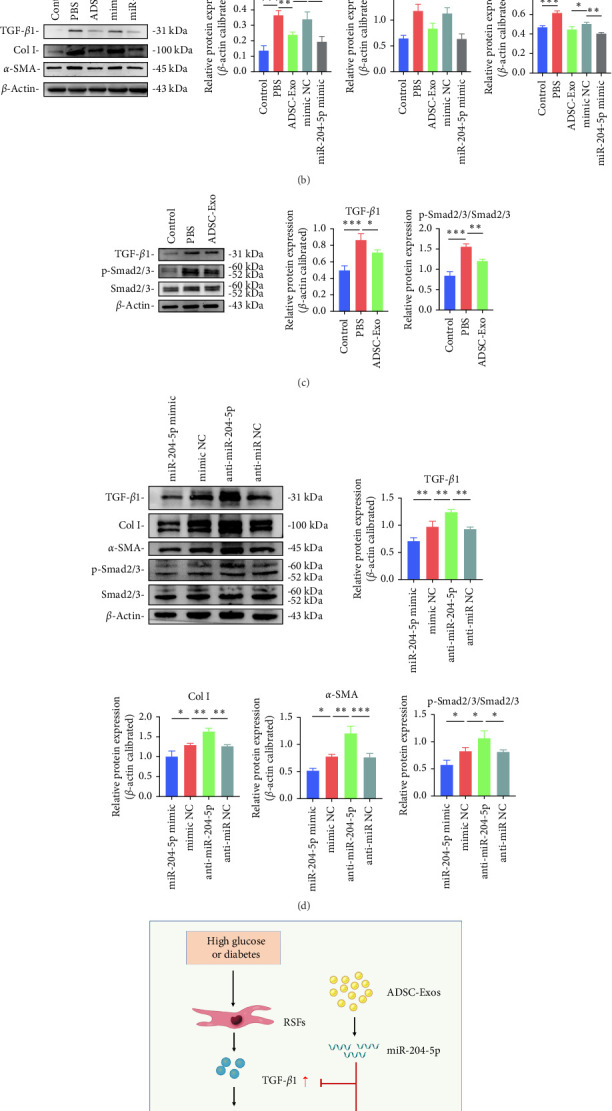
miR-204-5p regulates TGF-*β*1/Smad pathway. (A) RT-qPCR result of miR-204-5p. (B) Both ADSC-Exos and miR-204-5p mimic were able to reduce TGF-*β*1, Col I, and *α*-SMA. (C) ADSC-Exos demonstrated the ability to inhibit TGF-*β*1 and p-Smad2/3. (D) The miR-204-5p mimic could suppress TGF-*β*1/Smad signaling pathway activation, while anti-miR-204-5p had the opposite effect. (E) Schematic representation of miR-204 5p inhibition on scar formation. *⁣*^*∗*^*p* < 0.05, *⁣*^*∗∗*^*p* < 0.01, and *⁣*^*∗∗∗*^*p* < 0.001. ADSC-Exos, adipose-derived stem cell exosomes; *α*-SMA, alpha-smooth muscle actin.

Validation of high expression of miR-204-5p in ADSC-Exos was confirmed by RT-qPCR, with ADSCs serving as the control group (Figure 6A). We established control and HG groups and treated RSFs with PBS, ADSC-Exos, mimic NC, and miR-204-5p mimic, respectively. The western blot experiment demonstrated that miR-204-5p mimic, similar to ADSC-Exos, can reduce TGF-*β*1, collagen I, and *α*-SMA expression in an HG culture environment (Figure 6B). Smad2/3 phosphorylation is a key reaction step in the TGF-*β* signaling pathway. The results revealed that ADSC-Exos can reduce HG-induced phosphorylation of Smad2/3 (Figure 6C). We found that miR-204-5p mimic reduced TGF-*β*1 and p-Smad2/3 levels, as well as the downstream genes Col I and *α*-SMA in the TGF-*β*1/Smad pathway. Conversely, the anti-miR-204-5p significantly upregulated the levels of them (Figure 6D). These results demonstrate that miR-204-5p contained in ADSC-Exos can inhibit myofibroblast differentiation and the degree of fibrosis in the scar by targeting TGF-*β*1/Smad pathway (Figure 6E).

## 4. Discussion

We found that ADSC-Exos can rescue the proliferation and migration dysfunction in RSFs caused by HG. MiR-204-5p in ADSC-Exos inhibits the secretion of fibrosis-associated proteins via the TGF-*β*1/Smad pathway. In addition, subcutaneous injection of ADSC-Exos can promote diabetic wound healing and reduce scar formation. These results indicate that ADSC-Exos may be a potential nanotherapeutic agent for the treatment of difficult-to-heal wounds such as diabetic wounds.

Over the years, ADSCs have been studied as a research target for cell therapy in the field of tissue engineering and regenerative medicine. Multiple studies have demonstrated that ADSCs have a positive effect in tissue regeneration and wound repair. They can inhibit inflammation, increase vascular regeneration, promote re-epithelialization, and facilitate the regeneration of skin appendages [9, 33, 34]. It is worth noting that the therapeutic effect of stem cells is closely related to cell activity. Prolonged in vitro proliferation can affect the proliferation and differentiation ability of MSCs [35]. In contrast, exosomes do not require long-term ex vivo proliferation, making them easier to store and transport and exhibiting a more stable biological activity. Additionally, exosomes can directly act on target cells through phagocytosis, showing better biocompatibility [36]. To demonstrate the mechanism of action of ADSC-Exos on RSFs, we labeled ADSC-Exos with PKH26 dye and subsequently added them to the culture medium of RSFs. After 24 h, it was observed that ADSC-Exos appeared in the cytoplasm of RSFs, with a particular concentration around the cell nucleus. These results indicate that ADSC-Exos can exert their effects on RSFs by directly entering the cells.

To learn more about ADSC-Exos, we first performed in vitro experiments. We stimulated RSFs with HG to establish an HG-induced skin fibroblast injury model. Previous studies have found that HG can impair the proliferation and migration ability of fibroblasts. The mechanism may involve inflammation, oxidative stress, cellular senescence, and programed cell death induced by high sugar levels [17, 18, 37]. Our research results also indicate that an HG environment inhibits the proliferation and migration ability of RSFs, manifested as decreased cell proliferation activity, prolonged cell scratch wound healing time, reduced number of cells in Transwell migration experiments, and slow wound healing with decreased re-epithelialization. In comparison with the group with PBS, ADSC-Exos treatment can improve the proliferation and migration ability of RSFs, shorten the healing time of diabetic wounds, and promote re-epithelialization. In conclusion, these results indicate that ADSC-Exos improved wound healing. However, further exploration is needed in the future to understand the potential mechanisms involved.

The fibroblasts in the dermis are key cells for skin wound repair. During the proliferation and remodeling stages, fibroblasts cover the wound bed through proliferation and migration. Subsequently, they differentiate into myofibroblasts, promoting wound contraction and secreting a large amount of collagen, especially collagen I, which is the main component of scar tissue. After wound closure, myofibroblasts gradually disappear to prevent excessive scar formation [38, 39]. The activity state of fibroblasts and the degree of myofibroblast differentiation determine the speed of wound healing also the size of scars [40]. As mentioned earlier, the time frame of wound healing for different stages, including inflammation, proliferation, and tissue remodeling, is overlapping. The environment and process of diabetic wound healing are more complex compared to normal wounds. Research has shown that HG makes it difficult for M1 macrophages to differentiate into M2 macrophages [8]. M2 macrophages play a critical role in promoting myofibroblast differentiation and ECM production, and their deficiency can result in impaired wound healing. Contrary to the findings mentioned above, using single-cell sequencing experiments, Theocharidis et al. [41] found that people with diabetes who had healed wounds had a higher presence of M1 macrophages in their wounds, while those with nonhealing wounds had a higher presence of M2 cells. The conflicting viewpoints above indicate that the immune environment of diabetic wounds is extremely complex, and the activation of myofibroblasts may be a double-edged sword, the mechanism of which is not yet fully understood. Our data showed that the closure rate of diabetic wounds decreases, and the scars are longer. Granulation tissue proliferation in scar tissue 14 days after surgery is more pronounced. Masson staining and immunofluorescence staining showed more obvious differentiation of myoblasts and higher density of collagen fibers. Based on the results, the following points can be inferred. First, diabetic wounds heal slowly and have significant tissue fibrosis. Second, myofibroblasts should decrease in the late stages of wound healing, but the effects of HG increased TGF-*β*1 production, leading to the activation and prolonged presence of myofibroblasts in the wound bed. This leads to excessive deposition of type I collagen and scarring. Therefore, it is important to timely intervene to suppress fibrosis during healing, rather than treating scars after the wound has completely healed. Numerous studies have demonstrated that ADSC-Exos can promote wound healing and inhibit scar formation [42–44]. Our results are similar to the above-mentioned study. However, the molecular mechanism is still unclear, which intrigues us to explore further.

Exosomes contain a rich array of bioactive components, including miRNA. Multiple studies have demonstrated that exosomes derived from MSCs exert functional effects on target cells through the transfer of miRNA molecules [11, 45]. We analyzed the miRNA sequencing data (GSE92313) of ADSC-Exos reported by Zhang et al. [32], and combined with the miRwalk database, we found that miR-204-5p is highly expressed in ADSC-Exos and can target TGF-*β*1. This study utilized RT-qPCR to validate the high expression of miR-204-5p in ADSC-Exos and confirmed the targeting effect of miR-204-5p on TGF-*β*1 through dual luciferase reporter assay and western blot experiments. Previous studies have demonstrated that compared to normal fibroblasts, the expression levels of TGF-*β*1, phosphorylated Smad2, and Smad3 are elevated in scar fibroblasts. Therefore, the TGF-*β*1/Smad pathway is an important therapeutic target for scar formation [46]. MiR-204-5p has been reported to target TGF*β*R1 and TGF*β*R2, inhibiting the proliferation and invasion of synovial fibroblasts, thus alleviating collagen-induced arthritis [47]. Further research has also demonstrated that miR-204-5p can significantly inhibit TGF-*β*1-induced proliferation of tracheal smooth muscle cells and ECM deposition, thereby improving airway remodeling in asthma [48]. Previous studies have demonstrated that miR-204-5p is closely associated with the TGF-*β* signaling pathway. Building on this foundation, the study utilized ADSC-Exos and miR-204-5p mimic for simultaneous treatment of RSFs under HG conditions, revealing their similar effects. However, the phosphorylation of Smad2/3 proteins is a classic pathway in the TGF-*β*1 signaling cascade, which can induce myofibroblast differentiation and ECM deposition [49, 50]. Therefore, we hypothesize that miR-204-5p may influence the fibrotic level of dermal fibroblasts through the Smad2/3 signaling pathway. To investigate the effect of ADSC-Exos on the TGF-*β*1/Smad signaling pathway, western blot experiments were conducted. The results showed that ADSC-Exos inhibited the expression levels of TGF-*β*1 and the phosphorylation levels of Smad2/3. Transfection of RSFs with miR-204-5p mimic and inhibitor revealed that miR-204-5p mimic could suppress the TGF-*β*1/Smad signaling pathway and the expression of its downstream genes *α*-SMA and Col I, while the miR- 204-5p inhibitor produced opposite effects. It is evident that miR-204-5p exerts an inhibitory effect on the TGF-*β*1/Smad pathway by directly targeting TGF-*β*1. In conclusion, miR-204-5p in ADSC-Exos may serve as a potential target for preventing diabetic wound scar fibrosis.

Although our research has identified the positive role of ADSC-Exos in promoting diabetic wound healing and reducing scarring, as well as revealed the anti-fibrotic effect of miR-204-5p, there are still limitations in this study. First, we have not yet delved into the specific mechanisms of ADSC-Exos in promoting the proliferation and migration of RSFs under HG conditions. Future research should further explore their impact. Second, our model is based on fibroblasts and rats, considering only the HG factor without taking into account potential influencing factors such as pathogens, ischemia-hypoxia, and nerve damage. Therefore, future studies need to broaden the scope of research. Lastly, the data obtained when investigating wound healing at 14 days postoperatively has certain limitations. It is recommended to extend the observation period in future studies and conduct more diversified data collection to obtain a more comprehensive dataset.

## 5. Conclusion

In conclusion, this study showed that ADSC-Exos can improve the proliferation and migration of skin fibroblasts under the condition of HG, thereby accelerating the healing process of diabetic wounds. Furthermore, ADSC-Exos was found to inhibit myofibroblast differentiation and type I collagen deposition through miR-204-5p/TGF-*β*1/Smad pathway, ultimately reducing scar formation and improving the overall quality of wound healing.

## Data Availability

Data will be available upon request from the authors. The data that support the findings of this study are available from the corresponding author upon reasonable request.
